# Tuberculosis screening of asylum seekers in Finland, 2015–2016

**DOI:** 10.1186/s12889-020-09122-5

**Published:** 2020-06-19

**Authors:** Pirre E. Räisänen, Hanna Soini, Paula Tiittala, Olli Snellman, Petri Ruutu, J. Pekka Nuorti, Outi Lyytikäinen

**Affiliations:** 1 Health Sciences unit, Faculty of Social Sciences, Tampere University, P.O. Box 30, FI-00271 Helsinki, Finland; 2 Infectious Disease Control and Vaccinations Unit, Department of Health Security, Finnish Institute for Health and Welfare (THL), Helsinki, Finland; 3 Finnish Immigration Service, Helsinki, Finland

**Keywords:** Tuberculosis, Screening, Asylum seekers, Foreign-born

## Abstract

**Background:**

In Finland, asylum seekers from countries with high tuberculosis (TB) incidence (> 50/100,000 population/year) and those coming from a refugee camp or conflict area are eligible for TB screening. The aim of this study was to characterise the TB cases diagnosed during screening and estimate the yield of TB screening at the reception centres among asylum seekers, who arrived in Finland during 2015–2016.

**Methods:**

Voluntary screening conducted at reception centres included an interview and a chest X-ray. Data on TB screening and health status of asylum seekers was obtained from the reception centres’ national health register (HRS). To identify confirmed TB cases, the National Infectious Disease Register (NIDR) data of foreign-born cases during 2015–2016 were linked with HRS data. TB screening yield was defined as the percentage of TB cases identified among screened asylum seekers, stratified by country of origin.

**Results:**

During 2015–2016, a total of 38,134 asylum applications were received (57% were from Iraq, 16% from Afghanistan and 6% from Somalia) and 25,048 chest x-rays were performed. A total of 96 TB cases were reported to the NIDR among asylum seekers in 2015–2016; 94 (98%) of them had been screened. Screening identified 48 (50%) cases: 83% were male, 56% aged 18–34 years, 42% from Somalia, 27% from Afghanistan and 13% from Iraq. Furthermore, 92% had pulmonary TB, 61% were culture-confirmed and 44% asymptomatic. TB screening yield was 0.19% (48/25048) (95%CI, 0.14–0.25%) and it varied between 0 and 0.83% stratified by country of origin. Number needed to screen was 522.

**Conclusions:**

TB screening yield was higher as compared with data reported from other European countries conducting active screening among asylum seekers. Half of the TB cases among asylum seekers were first suspected in screening; 44% were asymptomatic. TB yield varied widely between asylum seekers from different geographic areas.

## Background

Tuberculosis (TB) remains a public health concern for low-incidence countries (< 10/100,000 population/year) primarily because of migration from high-incidence TB countries. Factors before and during migration, such as living at a refugee camp or conflict area, increase the risk of transmission [1]. During 2015 when 1.2 million asylum seekers entered Europe, Finland’s public health preparedness was tested: the screening of TB among asylum seekers had to be implemented in a short period of time [2]. In 2015, the third largest number of applications in Europe was received in Finland [3, 4], 615 applications per 100,000 inhabitants, compared with 1727 in Sweden, 653 in Norway and 537 in Germany [5].

Similar to other European countries, Finland has adopted active TB screening protocols for asylum seekers based on WHO recommendations [6]. The aim of screening for active TB is to find infectious cases at an early stage to protect the individual’s and the population health and by interrupting transmission. Despite recent recommendations by the European Centre for Disease Prevention and Control (ECDC), Finland does not currently screen for latent TB infections (LTBI) [7].

Published studies provide divergent information about yield of TB screening in European Union/European Economia Area (EU/EAA) countries [7–10]. EU/EEA countries use different screening strategies and settings: Belgium, Germany and Switzerland screen only asylum seekers and refugees, the Netherlands and Spain screen also other migrants arriving from high incidence areas, and Italy and the United Kingdom do not conduct systematic TB screenings for asylum seekers. Also, the timing and site of screening varies: pre-entry/pre-migration screening, port of arrival screening at the airport or harbour upon arrival, reception/holding/transit centre screening shortly after arrival in the country, and community post-arrival screening. In some countries TB screening is voluntary and other countries have a compulsory screening strategy [7–10]. Also, the yield of screening for active TB is presented in different ways: as percent or per 1000 asylum seekers. Screening prevalence rate (n/100000 individuals screened) is also used in parallel with yield [7].

Previous studies have reported yield of TB screening among migrants in EU/EEA countries [1, 8, 10], but only few studies have reported data on voluntary TB screening among asylum seekers in reception centres [11, 12]. The aim of this study was to characterise the TB cases diagnosed during screening and estimate the yield of TB screening at the reception centres among asylum seekers, who arrived in Finland during 2015–2016.

## Methods

### TB screening

All asylum seekers are given information on health, health services and infectious diseases at the reception centre [13]. Asylum seekers may also fill in a symptom-based health questionnaire on their own health including questions regarding TB, which is used to plan the urgency of the basic health check-up and infectious disease screening [2]. Attending the information session and completing the questionnaire was voluntary.

TB screening consists of two phases and an asylum seeker was considered to be screened if one of the two phases was complete. First, in the initial health check-up a nurse interviews asylum seeker about their risk factors of TB, such as symptoms (including extrapulmonary TB symptoms), having stayed at a refugee camp or in a conflict area, or previous history of TB. Second, chest X-ray (CXR) is performed within two weeks of arrival. CXR is offered to asylum seekers coming from countries that have a high incidence of TB (> 50/100,000 population/year) and those with other risk factors. CXR is voluntary but highly recommended for those asylum seekers who are asymptomatic but mandatory for those who have symptoms. In 2015–2016, CXR was performed by two nationally-contracted private healthcare providers. The number of asylum seekers who had CXRs performed was obtained from the immigration and healthcare procurement registers.

Information about the country of birth of the screened asylum seekers without TB diagnosis was not available due to non-systematic recording of health and screening information to HRS.

National Health Record System of the Reception Centres.

HRS is maintained by the Finnish Immigration Service, was introduced to the reception centres in 2014 and was comprehensively used in the beginning of 2016. HRS is used for maintaining the health and screening records of all asylum seekers regardless of whether they live in a reception centre or private housing. The following HRS data was collected on asylum seekers diagnosed with TB: name, date of birth, gender, nationality, date of interview and health check-up, date and findings of CXR, TB - related symptoms, date of symptom onset and further examinations performed.

### National Infectious Disease Register

All physicians and laboratories notify TB cases to the national infectious disease register (NIDR). The case definition for TB surveillance includes all cases confirmed by culture, sputum smear, nucleic acid amplification and/or histology [14]. A case is also reported based on physician’s decision to initiate full TB treatment due to clinical suspicion of TB, despite lack of laboratory confirmation. Each physician’s notification includes a unique national identifier, if available, name, date of birth, gender, country of birth, nationality, place of residence and treatment, date of symptom onset and diagnosis, diagnostic method and clinical presentation (pulmonary/extrapulmonary TB). The NIDR data is supplemented with details of the patient’s place of residence, country of birth, nationality and possible death from the National Population Information System, if the national identifier is available. The information about the asylum status is not notified in the NIDR data. Therefore, data on foreign-born TB cases (i.e. cases not born in Finland or unknown country of birth) notified to NIDR during 2015–2016, were linked to the HRS data by name, date of birth and origin to identify asylum seekers who had arrived in Finland during 2015–2016.

### Data analysis and statistics

Aggregated data of all asylum seekers by age group and country of origin who had arrived in Finland during 2015–2016 were obtained from the Finnish immigration service [15].

Overall TB yield was defined as the percentage of TB cases identified among screened asylum seekers. Aggregated data were used as denominator to calculate the yield by country of origin. The confidence intervals (CI) were calculated according to Wald [16]. The number needed to screen (NNS) was calculated as the number of persons screened divided by the number of TB cases found in screening. Cross-tabulation was used to analyse the data. The data analysis was performed using Microsoft Excel 2010 (Microsoft, Redmont, Washington, USA) and IBM SPSS statistics 25.0 (SPSS Inc., USA).

## Results

From January 1, 2015 through December 31, 2016, a total of 38,134 asylum seekers arrived to Finland (Table 1): most were men (80%) and 18–34 years of age (60%). Over 80% of asylum seekers came from Iraq, Afghanistan, Somalia or Syria. A total of 34,998 asylum seekers were eligible for CXR screening and 25,048 CXRs were performed (coverage, 72%; 95% CI, 71.1–72.0%) [2].

**Table 1:** Characteristics of asylum seekers, TB cases among asylum seekers, TB cases diagnosed based on screening and yield of TB cases diagnosed based on screening, 2015–2016, Finland

		All asylum seekers; n (%),	All TB cases among asylum seekers; n (%),	TB cases diagnosed based on screening; n (%)	Yield of TB cases diagnosed based on screening; (%)
		*n* = 38,134	*n* = 96	*n* = 48	0.19 (48/25048)
Men^a^		30,122 (79)	71 (74)	40 (83)	
Age group^b^	0–13	5669 (15)	3 (3)	1 (2)	
	14–17	3740 (10)	13 (13.5)	9 (19)	
	18–34	22,397 (59)	65 (68)	27 (56)	
	35–64	6024 (16)	13 (13.5)	11 (23)	
	65 or above	114 (0.3)	2 (2)	0	
Origin^c^	Iraq	21,731 (57)	11 (11)	6 (12.5)	0.028^e^
	Afghanistan^d^	5968 (16)	17 (18)	13 (27)	0.22^e^
	Somalia^d^	2413 (6)	49 (51)	20 (42)	0.83^e^
	Syria	1479 (4)	2 (2)	0	0
	Other	6493 (17)	17 (18)	9 (19)	NA
Pulmonary TB			71 (74)	44 (91.7)	
MDR-TB			8 (8.3)	4 (8.3)	

^a^ For 53 (0.1%) asylum seekers, information on sex was missing

^b^ For all asylum seekers, age at the time of immigration; for TB cases age at the time of diagnosis. Of all the asylum seekers, 190 (0,5%) had unknown age

^c^ Origin is based on country of birth, and if not available, on nationality. Of all the asylum seekers, 50 (0,1%) had unknown origin

^d^ Incidence rate > 50/100000 population in 2015–2016

^e^ Aggregated data were used as denominator to calculate the yield by country of origin

**Fig. 1:**
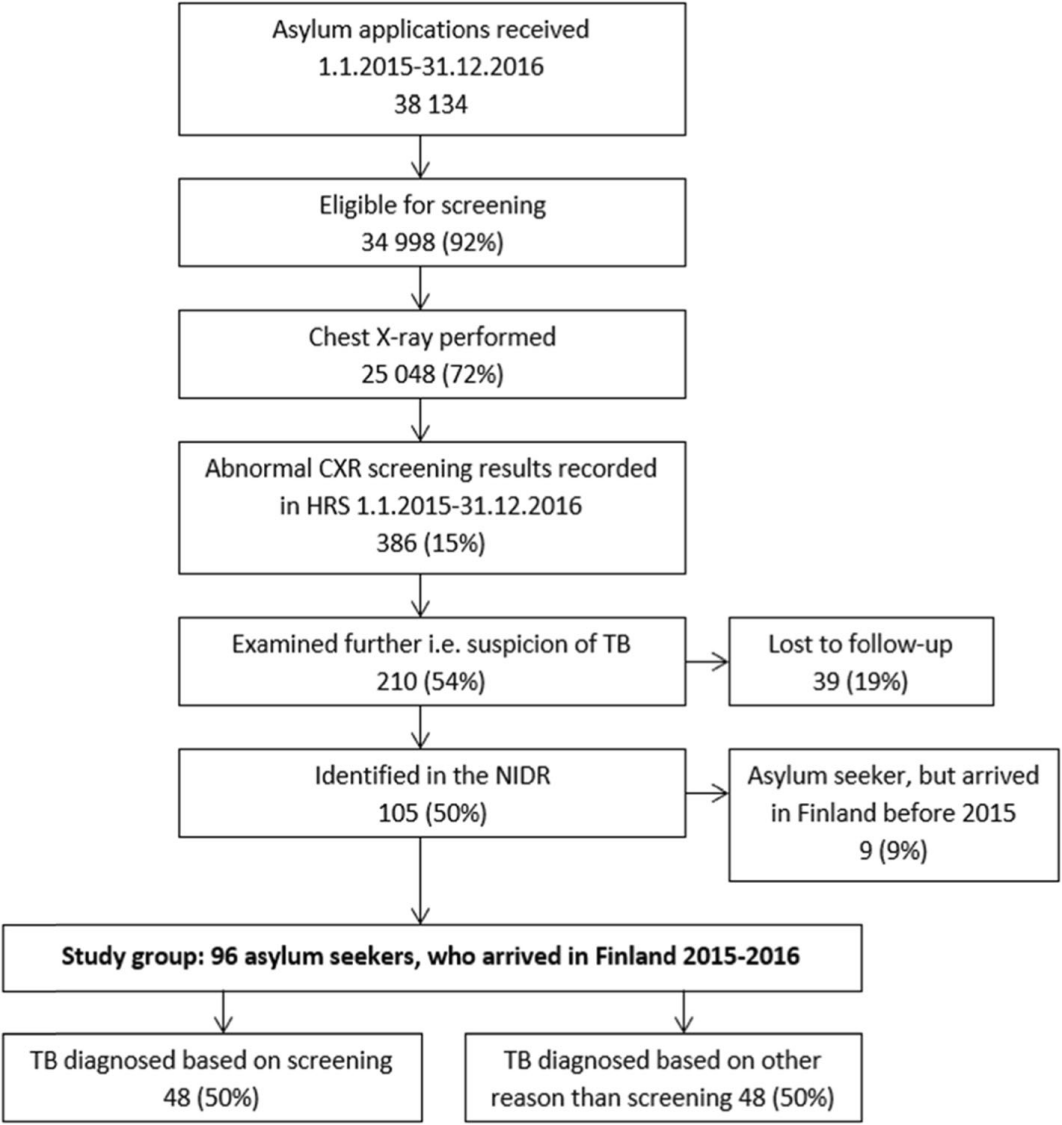
Flow chart of the screening process and study groups

Altogether 386 abnormal screening results were recorded in the HRS; 210 (54%) were examined further, i.e. there was suspicion of TB, and 39 (18.6%) of them were lost to follow-up (Fig. 1). We identified 105 asylum seekers in the NIDR who had been diagnosed with TB in 2015–2016; 9 asylum seekers had arrived in Finland before 2015 and therefore were excluded from the analysis. A total of 96 asylum seekers, who had arrived in Finland during 2015–2016, received a diagnosis of TB during 2015–2016, and of them 48 (50%) were diagnosed based on screening.

Of the 48 TB cases diagnosed based on screening, 83% were male and the median age was 25 years (range, 3–62) (Table 1). The most common country of origin was Somalia (40%), Afghanistan (27%) and Iraq (12.5%). Altogether 41 (85%) had attended the health information session and/or initial health check-up; the attendance was not documented in 7 (15%) cases. All 48 cases had undergone screening CXR; 40 (83%) had abnormal findings and in 8 (17%) cases detailed CXR results were not documented. Pulmonary TB was diagnosed in 44 (92%) cases; 27 (61%) were culture-confirmed, 27 (61%) had symptoms, and 7 (16%) were sputum smear positive. The date of CXR was available for 41 cases; the median time from CXR screening to the time of diagnosis was 18 days (range 1–376 days). Two of the cases had a long diagnostic delay; one case was due to pregnancy (180 days); in the other case (376 days) the reason was unknown.

Of the 48 TB cases diagnosed based on other processes than screening, 73% were male and the median age was 22 years (range, 5–82). The most common country of origin was Somalia (56%), Iraq (8%) and Afghanistan (6%). In 40 (83%) cases TB suspicion arose because of symptoms which appeared after screening, for 5 (10%) cases the reason of TB suspicion was unknown, 2 (4%) were found before screening and 1 (2%) in contact tracing. Altogether 36 (75%) had attended the health information session and/or initial health check-up; the attendance was not documented in 12 (25%) cases. A total of 46 (96%) cases had undergone screening CXR; 5 (11%) had abnormal findings and 41 (89%) cases’ detailed CXR results were not documented. Pulmonary TB was diagnosed in 27 (56%) cases; 24 (89%) were culture-confirmed, 24 (89%) had symptoms, and 6 (22%) were sputum smear positive. The date of CXR was available for 14 cases; the median time from CXR screening to the time of diagnosis was 230 days (range 97–395 days).

TB yield among individuals screened was 0.19% (95%CI, 0.14–0.25%) and NNS 522. When assuming that all asylum seekers from the same country of origin were screened, TB yield ranged from 0 to 0.83% by country of origin, being highest for Somalia (Table 1). Accordingly, screening prevalence rate among asylum seekers was 191/100000 and ranged from 0 to 828 cases/100000 by country of origin.

## Discussion

We evaluated the screening of active TB in asylum seekers arriving in Finland during the large influx in 2015–2016. A total of 96 TB cases were diagnosed among asylum seekers and reported to the NIDR during this time period. Half of them were first suspected in screening and over 40% of these cases were asymptomatic. Pulmonary TB was more common among those diagnosed based on screening. There was a 40-fold difference in TB prevalence among asylum seekers from different geographic regions. For those originating from Iraq, the prevalence of TB was low. On the other hand, individuals of Somali background, who constituted only 6 % of the asylum seekers, constituted half of the TB cases.

During the 2015–2016 influx of asylum seekers, other European countries also conducted TB screening at reception centres. We found that the yield in Finland was higher than the average yield reported from other European countries (0.19% vs. 0.12%) [8]. That said, the screening prevalence rate of TB (191/100000) and NNS (522) in Finland differed greatly from Italy and Germany [1, 12]. In Italy, post-entry screening prevalence rate of TB in asylum seekers was 535/100000 and NNS 187, among the highest in Europe. In Italy, most of the asylum seekers (82%) originated from very high TB prevalence countries in Africa, contributing to the higher screening yield. In Germany, where TB screening is mandatory, the prevalence was 347/100000 and NNS 288. This variability may be associated with differences in countries of origin and migration routes of asylum seekers as well as implementation of screening policies [1, 12].

Somalis represented over half of all TB cases in asylum seekers and over 40% of TB cases found by screening. In our previous studies [17, 18], persons of Somali origin accounted for approximately 30% of TB cases among migrants in Finland. Furthermore, it is also of note that 76% of all TB cases in persons from Afghanistan were found in screening, suggesting that Afghan asylum seekers may have had advanced TB disease at the time of arrival to Finland.

The delay from CXR to TB diagnosis ranged from 1 to 376 days (median, 18 days), which is in line with the findings in a recent systematic review and meta-analysis (the median diagnostic delay ranged from 30 to 366.5 days) [19]. In a previous Finnish study [2] of the same study population, the median delay from arrival in Finland to performing CXR for an asylum seeker was 43 days for children and 74 days for adults. Adding up the previous finding from arrival to CXR with our finding of median delay from CXR to TB diagnosis (18 days), the total median delay from arrival in Finland to TB diagnosis was estimated to be two to three months.

There are several limitations that should be considered when interpreting our findings. First, during 2015, the national health record system (HRS) was not fully implemented at all reception centres. Therefore, all health records were not entered systematically in the HRS. In addition, all of the screened asylees’ backgrounds or countries of birth were not recorded which may have led to incomplete electronic records, too small denominator and potential underreporting of screening and yield. Second, all asylum seekers did not have the unique national identifier, because they were not assigned it during the asylum process. Furthermore, due to unavailability of national identifier, misspelling of asylum seekers’ names and varying dates of birth in different databases caused difficulties in linking HRS and NIDR. Taken together, this means that the numerator in the yield calculation might be too large, leading to overestimation of screening yield. Third, it is possible that TB was not always detected by CXR at the time of screening; almost 20% of the asylum seekers with abnormal screening results were lost to follow-up before further examinations. Finally, we were unable to assess the sensitivity and specificity of screening as it was not possible to detect false-positive cases, since abnormal CXR result due to other diagnoses than TB were also documented in the HRS CXR results section. The reporting system could be improved by assigning the unique national identifier to asylum seekers at the border when arriving to Finland. This would make electronic screening documentation available at all healthcare centres and reduce loss to follow-up. Also, the national identifier would help linking records between registers. Mandatory screening of all asylum seekers, however, has not been found cost-effective in the Finnish context [2, 18].

## Conclusions

In conclusion, the yield of TB screening was not as high as expected, since the asylum seekers during 2015–2016 originated more often from conflict areas with a low or moderate TB incidence than countries with high TB incidence. Even if the current guidelines seem adequate for screening active TB in Finland, interventions with screening and treatment of latent TB infection, as recommended by the ECDC, could be considered [7]. Careful prioritization of resources and TB screening criteria, combined with efficient reporting of data, are needed when there is an unusual pressure on limited services.

## Data Availability

The datasets generated and/or analysed during the current study are not publicly available due to possibility of recognition of a patient even though data does not include personal level data but are available from the corresponding author on reasonable request.

## Abbreviations

CI: Confidence interval
CXR: Chest X-ray
ECDC: European Centre for Disease Prevention and Control
EU/EEA: European Union/European Economic Area
HRS: National Health record system of asylum seekers
LTBI: Latent Tuberculosis Infection
Migri: Finnish Immigration Service
NIDR: National Infectious Disease Register
NNS: Number Needed to Screen
TB: Tuberculosis
WHO: World Health Organisation
